# Individual interventions, collective lessons: Developing mid-range theory on women’s groups to improve health

**DOI:** 10.7189/jogh.14.04152

**Published:** 2024-08-16

**Authors:** Sapna Desai, Neha Kumar, Lu Gram, Avishek Hazra, Kaliat Ammu Sanyal, Sharmada Sivaram, Nirmala Nair, Rajani Ved, Audrey Prost

**Affiliations:** 1 Population Council Institute, New Delhi, India; 2 International Food Policy Research Institute, Washington DC, USA; 3 UCL Institute for Global Health, London, UK; 4 Population Council Consulting, New Delhi, India; 5 Independent Researcher, New Delhi, India; 6 Ekjut, Jharkhand, India; 7 Former ED, National Health Systems Resource Centre, New Delhi, India

## Abstract

**Background:**

Interventions with women’s groups have been widely implemented to improve health outcomes in low- and middle-income settings, particularly India. While there is a large evidence base on the effectiveness of single interventions, it is challenging to predict whether a women’s group intervention delivered in one setting can be expected to work in another.

**Methods:**

We applied realist principles to develop and refine a mid-range theory on the effectiveness of women’s groups interventions, summarised key lessons for implementation, and reflected on the process. We synthesised primary data from several interventions in India, a systematic review, and an analysis of behaviour change techniques. We developed mid-range theories across three areas: maternal and newborn health, nutrition, and violence against women, as well as an overarching mid-range theory on how women’s groups can improve health.

**Results:**

Our overarching mid-range theory suggested that effective interventions should: build group or community capabilities; focus on health outcomes relevant to group members; and approach health issues modifiable through women’s individual or collective actions. We identified four key lessons for future interventions with women’s groups, including the importance of skilled and remunerated facilitation, sufficient intensity, supply-side strengthening, and the need to adapt delivery during scale up while maintaining fidelity to intervention theory.

**Conclusions:**

Our experience demonstrated the feasibility of developing mid-range theory from a combination of evidence and insights from practice. It also underscored the importance of community engagement and ongoing research to ‘thicken’ mid-range theories to design effective and scalable women’s groups interventions in India and similar settings.

Public health policy in India promotes community engagement as an important strategy to improve maternal and child health and, increasingly, comprehensive primary health care [1]. Two major policy initiatives in India engage women’s groups in health interventions: the National Health Mission (NHM) engages health workers (ASHAs) to facilitate participatory learning and action (PLA) cycles with women in some states, leading to substantial improvements in neonatal mortality [2]. The National Rural Livelihoods Mission (NRLM) engages women’s self-help groups (SHGs), who are involved in savings, credit and livelihoods activities, in interventions that include information dissemination on nutrition, maternal health, and linkages with non-communicable disease programmes [3,4].

We previously conducted a systematic review of 99 studies to examine the effectiveness of women’s groups interventions on women and children’s health in India. Interventions have improved health outcomes in several domains such as maternal and newborn health, vector-borne disease control, and sexual and reproductive health [5]. However, we found that this evidence base had several limitations. First, existing women’s group interventions varied widely, including in the type of health outcomes they addressed, and the evidence base did not provide sufficient information to predict whether a women’s group intervention developed for one type of health outcome might work for another [6,7]. Second, the existing literature did not provide much information on key principles to maintain impacts at scale, as most efficacy studies have been conducted on small to medium-scale interventions. Addressing these limitations is important given the increasing emphasis placed on community engagement through women’s groups to improve health outcomes in India and beyond [8].

Recent work in implementation science has called for greater use of mid-range theorising – a foundational approach to theory construction in sociology – to address the challenge of developing theory out of heterogenous evidence bases [9,10]. Mid-range theories sit between working hypotheses about why events unfold in a certain way in a given context and broader theory [11,12]. They need to be specific enough to clearly explain observed phenomena, such as how health outcomes might improve through a given intervention, but general enough to hold across a range of settings [13]. A key challenge with using mid-range theories is moving up and down the ‘ladder of abstraction’ spanning context-specific programme theories of change and mid-range theories [14]. Mid-range theories that have clear, traceable foundations in empirical data and identify key causal steps to impact are likely to provide a better foundation for abstraction and guide to transferability.

Bonnell et al. (2023) recently highlighted the usefulness of mid-range theory in applying lessons from complex interventions [10]. Yet there are few descriptions of the process of developing mid-range theory in global public health. Anderson et al. (2023) drew from previous evaluations to highlight how clear theories of change can aid learning about women’s groups interventions, but also noted the difficulties of incorporating contextual factors so that learnings from one context can inform another [15]. this study collected and synthesised primary and secondary data to develop a mid-range theory to predict how interventions with women’s groups can improve women and children’s health in multiple settings. Using our mid-range theory, we identify key implementation science lessons regarding women’s groups interventions and illustrate the process of theory development as a worked example for others aiming to apply a similar approach.

## METHODS

### Mid-range theory development process

Our process followed three phases. First, we developed preliminary mid-range theories based on three empirical sources: i) a mixed-methods systematic review published in 2020 that included 44 quantitative impact evaluations and 55 non-experimental (quantitative and qualitative) studies that examined effects, enablers and barriers related to women’s groups interventions in India [5], through which we identified overarching principles for women’s groups to achieve health outcomes; ii) an analysis of social and behaviour change theories relevant to women’s groups, which we previously used to develop a published typology of approaches [7]; iii) and a mapping of social and behaviour change techniques to interventions, using a taxonomy developed by Kok et al. to categorise 14 types of techniques that aim to improve individual knowledge, capacity and skills or social and environmental conditions, an application of which was published in our previous systematic review [5,16]. During this phase, we also consulted over thirty implementers and stakeholders, including through a roundtable in 2019 and visits to six programmes across different states in India and meetings with experts in women’s groups between 2020–2022.

We developed a broad mid-range theory on women’s groups and health, along with three domain-specific theories in maternal and newborn health, nutrition, and violence against women (Table S1 in the **Online Supplementary Document**). We used four criteria to choose these domains: sufficient evidence available with impact and/or process evaluation data; implementation and evidence in at least two different contexts in India; priority areas for women’s group implementers, as indicated by engagement with government and community-led or non-governmental programmes; and previous engagement in the domain area amongst the study team. We intentionally worked as a consortium of individuals who have engaged with different types of women’s groups programmes, both to ensure we could review each with similar insight as well as to provide a balanced, objective view across programmes through reflexive discussions within the team and through workshops with external experts.

Second, we developed in-depth case studies in each domain to contextualise the initial mid-range theories using a ‘thicker’ set of programme-specific details. **Table 1** describes the programmes used as case studies. Most of the interventions reviewed in these case studies were based in rural areas of three states of India (Bihar, Jharkhand, and Odisha) that face similar challenges related to improving health outcomes amongst women and children. The urban programme in Mumbai, Maharashtra – India’s largest metropolis – works with the urban poor, particularly migrants. The interventions implemented in each state represent a range of types of women’s groups and approaches (**Box 1**).

**Table 1:** Case study sites

Domain	Programme	Implementer	Geography	Data sources	Description
**Maternal and newborn health**	FLAG (Facilitated Learning & Action Groups), 2015–20	Government of Jharkhand and Ekjut	Jharkhand	Impact evaluation [4], process evaluation	PLA cycles facilitated through ASHAs and ASHA facilitators (supervisors) to improve birth outcomes
	JTSP (JEEViKA Technical Support Program), 2015–ongoing	Bihar Rural Livelihoods Promotion Society (JEEViKA)	Bihar	Impact evaluation, systems assessment, 10 Key Informant Interviews (KIIs)	Behaviour change communication interventions around Maternal and Child Health and Nutrition through women’s SHGs
**Nutrition**	JEEViKA – Multisectoral convergence pilot, 2016 – 2018	Bihar Rural Livelihoods Promotion Society	Bihar	Impact evaluation [17], process evaluation	Behaviour change communication focused on maternal and child nutrition delivered by a community mobiliser at bi-monthly SHG meetings
	WINGS (Women Improving Nutrition through Group-based Strategies), 2015–20	PRADAN and Public Health Resource Society	Odisha, Chhattisgarh, Jharkhand, West Bengal, Madhya Pradesh	Impact evaluation [18]	Provision of health and nutrition messages at monthly SHG meetings through a dedicated volunteer cadre and integrated into pre-existing agriculture activities
	UPAVAN (Upscaling Participatory Action and Videos for Agriculture and Nutrition), 2015–20	Voluntary Association for Rural Reconstruction and Appropriate Technology coordinated by Digital Green, with technical assistance by Ekjut	Odisha	Impact evaluation, process evaluation [19]	Four arms that included: (1) women’s groups viewing and discussing videos on nutrition-sensitive agriculture (NSA) practices, and home follow-up visits to encourage adoption of new practices; (2) women’s groups viewing and discussing videos on NSA and nutrition-specific practices, with follow-up visits; and (3) women’s groups viewing and discussing videos on NSA and nutrition-specific practices combined with PLA meeting cycle, with follow-up visits and (4) a control arm.
	AAM (Action Against Malnutrition), 2012–17	Public Health Resource Society, Ekjut, CINI (Child in Need Institute), CHAUPAL, IDEA	Jharkhand, Odisha, Bihar, Chhattisgarh	Impact evaluation [20]	Civil society-led, community-based initiative to supplement the efforts of frontline health and nutrition workers through monthly PLA meetings with women’s groups, counselling through home visits, and crèches for children aged 6 mo to 3 y combined with PLA meetings and home visits.
**Violence against women**	SNEHA-TARA interventions to prevent violence against women and girls in informal settlements in Mumbai, 2018–2023	SNEHA (Society for Nutrition, Education & Health Action)	Maharashtra	Process evaluation [21]	Large-scale violence prevention programme in informal settlements in Mumbai combining community mobilisation through community groups with services for survivors of violence.
	SWAYAM (Strengthening Women’s Institutions for Agency and Empowerment), 2019–	IWWAGE (Initiative for What Works to Advance Women and Girls in the Economy) in collaboration National/State Rural Livelihoods Missions and 4 civil society organisations	Chhattisgarh, Madhya Pradesh, Jharkhand, Odisha	10 key informant interviews, qualitative process monitoring and reports [22]	Combination of consciousness-raising activities and institutional strengthening with SHGs and federations

Box 1 Types of women’s groups and approaches

Our systematic review on women’s groups to improve women and children’s health in India identified four major types of groups in this context:

Open groups formed through community mobilisation: groups formed to work on shared health issues, typically through facilitated, participatory learning and action cycles with community members.

Self-help groups: closed groups of 10–15 women that focus on livelihoods and financial security, many of which ‘layer’ on health activities. Self-help groups in the NRLM are federated at higher levels, starting with the Village Organization and Cluster Level Federation.

Community-based women’s groups: open or closed groups formed towards broad development objectives and social solidarity amongst women. These are of varying sizes and approaches, and work on health amongst other issues.

Special population groups: open or closed groups formed in a specific population, such as mothers’ groups or sex workers collectives, towards social solidarity and shared objectives [5].

Each case study examined: i) implementation approach, ii) enablers/barriers to implementation, and iii) the validity of the mid-range theory components. In addition, we examined specific questions for each domain that could relate to scale and transferability. Data sources included project documents and data from ongoing and recently completed impact and process evaluations. For two intervention sites where earlier or ongoing process data were not available, we conducted additional interviews with 14 key informants, all of whom were programme implementers working directly with women’s groups to explore questions that arose and to integrate their perspectives on key enablers and barriers to achieving health outcomes. In the third and final stage, we refined the overarching and domain-specific mid-range theories through mapping each intervention onto the mid-range theories, identified consistency and departures to add or remove contextual considerations and mechanisms, and reflected on the process.

## RESULTS

### Summary of mid-range theories

We developed an overarching mid-range theory, along with domain-specific theories, to explain how women’s groups interventions improve health outcomes (**Box 2**, Table S1 in the **Online Supplementary Document**).

Box 2 Mid-range theory

Engaging with women’s groups that build group or community capabilities and with sufficient intensity and facilitation can improve health outcomes that are relevant to the group and modifiable through individual or collective action.

Our overarching theory posits that interventions focused on building the capabilities of groups and communities through activities such as joint problem identification or group-based collective action operate differently than interventions that use the group as a platform for individual-level information transfer. Deciding whether to build the capabilities of groups only, or of both groups and the wider community will depend on the health outcome being addressed and contextual features, as discussed in-depth elsewhere [7]. Intervention intensity – which includes the proportion of relevant women engaged, duration, length and coverage of meetings and interactions–is an important marker to understand potential effectiveness [23].

We further identified principles that should influence the choice of health outcome: a health issue relevant to most women in the community, such as neonatal mortality in rural high-mortality settings rather than in a low-mortality urban context; ensuring that most group members are directly affected by this outcome; and that the outcome is modifiable through group or individual actions without a major supply side intervention beyond groups’ control. Finally, we identified four key lessons from our mid-range theories.

### Key lessons from mid-range theories

Lesson 1: Facilitators for women’s groups need to be appropriately trained and incentivised

Appropriate and adequate training and support is necessary. Many interventions relied upon the transmission of knowledge from programme trainers to facilitators, group members, and community members at large. Information loss presented a considerable challenge for these types of interventions, especially when facilitators had other tasks besides from conducting meetings. An in-depth evaluation of training of peer-educators revealed that receiving at least three trainings/refreshers increased the likelihood of facilitators retaining knowledge [24]. Skill-based training, such as explaining how to conduct a referral, emerged as an important approach – as did material support such as identification cards and field-based or on-the-job support and training.

Incentives for facilitators matter. In most sites, staff were paid honoraria, but there was variation in timeliness, adequacy and regularity of remuneration. In one intervention focused on reducing violence against women, three out of four intervention sites had ‘gender champions’ recruited to conduct training sessions on gender for members of the local village organisation, either through a fixed salary or per-training fees. An implementer observed that non-paid volunteers did not command the same respect in the community as those with standard remuneration, which in turn influenced their effectiveness. The programme eventually changed strategy towards fixed remuneration to improve outreach.

Lesson 2: Sufficient intensity is required to improve health outcomes

Intensity of exposure matters. Process data from surveys, meeting observations, and process evaluations across eight maternal and child health and nutrition interventions with SHGs indicated that most had limited intensity: health and nutrition meetings with SHGs lasted for approximately 27 minutes per month, and around 30–40% of women with young children were reached through home visits and about 20% through community events [23]. Other interventions used a PLA approach in group settings supplemented with home visits. Only the latter reported improvements in children’s nutritional status, including wasting and underweight [20].

Reaching the right community members matters. In interventions with closed SHGs, messages were delivered to women who were on average, 38 years old. Information on maternal and child nutrition was not directly relevant to these older SHG members, but may have been relevant for other household members, or other households within the community; the programme design assumed that older women would disseminate these messages to others. However, evaluations thus far suggest that programme reach to non-SHG members was limited [23]. Interventions with open groups that practised PLA were designed to include women who stood to benefit the most (e.g. pregnant women) but were also open to other concerned and influential women (e.g. their mothers-in-law).

In an intervention to address violence against women, implementers often only engaged members of SHGs’ federated village organisations rather than mobilising members of the village at large. Identifying survivors of violence is challenging, and survivors generally preferred turning to women they trust. Implementers noted that comparatively few women had approached them for help with gender-based violence compared to the number of women approaching them for help with access to government entitlements, a much less sensitive issue.

Lesson 3: Not all health outcomes can be improved without significant supply-side strengthening

Changing supply-dependent behaviours require systems linkages. Unless resource and supply-side constraints are addressed, it is unrealistic to expect demand-side interventions to have large impacts. Programme evaluations indicated that providing information to improve health and nutrition in SHGs did not significantly change supply-dependent behaviours such as consuming iron tablets or receiving postnatal visits, which require supplies and health providers. Evaluations noted a substantial gap in coordination and linkages between SHG programmes and frontline health workers at the ground level [25]. Adequate funding for supply-side interventions and facilitators who were linked to health services was an important design component of effective interventions.

Supply-side interventions must be sufficient. All interventions to address violence against women complemented demand-side group interventions with supply-side interventions, whether through physical or online counselling centres or training of local village organisations to directly provide counselling themselves. However, there were indications that further supply-side strengthening was needed. Women staffing counselling centres in one programme were expected to support individual survivors of violence by engaging with the police or the panchayat on their behalf. Lack of engagement with these institutions created barriers to cooperation and ultimately resulted in programme staff conducting their own community arbitrations without the involvement of other authorities.

Lesson 4: Scaling up requires adapting intervention delivery while maintaining fidelity to intervention theory

Interventions may need to be adapted to maintain fidelity at scale. In the domain of maternal and newborn health, interventions that were efficacious in small to medium-scale studies needed to be adapted to maintain fidelity to intervention theory at scale. For example, an intervention with ASHAs facilitating women’s groups practising PLA successfully reduced neonatal mortality in rural areas of Jharkhand and Odisha (2009–2012) [26]. Scaling up the intervention to Jharkhand’s 24 districts (2015–2020) while maintaining fidelity to core principles – collective problem-identification and -solving, inclusion of the poorest, a ratio of one group per 500–1000 people – required significant changes in intervention delivery: the National Health Mission (Jharkhand) in partnership with Ekjut, involved ASHA supervisors as women’s group facilitators in addition to ASHAs, reconfigured ASHA catchment areas to make their facilitation and other work manageable, developed novel training and supervision mechanisms, and made efforts towards the timely payment of incentives [2].

Similarly, Bihar’s rural livelihoods mission scaled up an intervention integrating maternal, newborn and child health interventions into existing SHGs with external technical support. Over time, the programme coverage and implementation strategies underwent substantial changes, with new human resource structures, increased use of home visits to discuss advice given in groups, as well as shifts in programme focus to nutrition and sanitation. Our interviews and documentary sources found that while staff and cadres’ capacities and participation in planning or review meetings improved over time, increased workload has been a concern.

The shifts in delivery modes responded to health system opportunities and constraints in each setting, but had different effects in these two programmes. Intervention effects on neonatal mortality were maintained at similar levels to those seen in efficacy trials in Jharkhand, despite some changes in intervention content, possibly because intensity of coverage and focus on maternal and newborn health remained high. Effects on maternal and newborn health outcomes in Bihar are yet to be published, but low intensity of exposure to MNH training suggests the possibility of lower effects on behavioural outcomes, and effects on mortality were not measured.

## DISCUSSION

We developed a mid-range theory of how interventions with women’s groups can improve women and children’s health. Our theory posits that engagement of sufficient intensity with women’s groups that build group or community capabilities can improve health outcomes relevant to the group through individual or collective action.

Specific features of the theory highlight areas to which implementers must pay specific attention [10]. First, we highlight the need for appropriate training, supervision and incentivisation of group facilitators. A substantial theoretical and empirical literature explores community health worker motivation and performance in programmes engaging community members individually [27-29]. To our knowledge, less has been written on group facilitators whose performance on engaging group members in reflection and action may be harder to define. However, this does mean that group-based programmes can afford to overlook issues of training, supervision, and incentivisation.

Second, our mid-range theory explicitly emphasises the need for sufficient intervention intensity. ‘Intensity’ is often left unmentioned or implicit in theories of health promotion through women’s groups [23,30]. We encourage future evaluators to adopt common reporting indicators for intensity to allow for better comparison between interventions [6]. Ambiguity over the meanings and relevant indicators of ‘intensity’ in group-based programmes risk encouraging policymakers to fund less costly, but also less effective, low-intensity interventions.

Third, significant supply-side strengthening was required for interventions for some health outcomes. Public health researchers have long cautioned against undue optimism in the power of community participation to transform health outcomes without adequate support from the broader health system [31,32]. By examining multiple interventions across multiple health domains – rather than evaluate a single intervention using a single trial – we found evidence that improving nutrition and addressing violence against women may be more supply-dependent than reducing neonatal mortality in settings where mortality rates are high. This is because improving nutrition and addressing violence requires addressing families’ food insecurity and offering services to support survivors of violence, whereas small changes to essential newborn care practices at home can reduce neonatal mortality in settings where it is high. Women’s groups are not a generalised policy tool to tackle any health outcome. Rather the design of women’s group interventions needs to be specifically tailored to each health outcome in question.

Finally, we found that scale up required intervention delivery to be adapted while maintaining fidelity to intervention theory. ‘Fidelity to theory’ may involve drastically different design choices for the same intervention at different scales of implementation. Local decisions about implementing interventions evaluated as effective elsewhere in new contexts can be supported by mapping out how an intervention was implemented and contextual factors that supported or derailed it, using an approach proposed by Cartwright et al. [33,34]. Maintaining fidelity to these intervention principles and support factors while scaling up can make the difference between success and failure.

### Implications for programmes

The fact that many interventions with women’s groups fail to produce desired effects on health reflects a need for programme planners to map causal principles, support factors and derailers in greater detail. The mid-range theory could have helped predict that interventions using SHGs to deliver behaviour change messages to older women about infant and young child feeding in food insecure contexts, without sufficiently intense supply-side interventions, would be unlikely to affect children’s nutritional status [18]. Drawing from the mid-range theory could also emphasise the importance of adaptation when scaling up to inform the ongoing expansion of ASHA-led and facilitated PLA interventions. Further, our research underscores the need for more work that engages women directly to understand which areas of health and well-being they would most want to address in the context of group interventions. The ‘nothing about us without us’ mantra applies in this as in all other health policy contexts [35].

#### Strengths and limitations

The key strength of this effort to build a mid-range theory was our use of multiple primary and secondary data sources. Our analysis would have been improved by additional direct observation of interventions, which was not possible due to COVID-19 restrictions. We also identified limitations inherent to using mid-range theory; **Box 3** details our reflections on the process of mid-range theory development, its strengths and limitations. Our reflections underscored that, while mid-range theory offers an approach to distilling key intervention characteristics, its application requires an understanding of context, the ‘how’ of implementation and recognition of principles of community participation that underlie effective approaches to working with women’s groups.

Box 3 Reflecting on the process of mid-range theory development

We found the process of mid-range theory development useful to clarify relationships between contexts, intervention mechanisms and outcomes using existing data, but also encountered some challenges.

First, the process often felt circular, as we developed and refined mid-range theories based on our knowledge of the effectiveness and found limitations of interventions. We tried to mitigate this through engaging with diverse types of actors to contribute to and review our findings. Our experience underscores the importance of a multi-disciplinary consortium when undertaking such an exercise to ensure variation in experience and insights.

Second, many of the elements of our overarching theory seemed so obvious that we felt few implementers would disagree with them or find them novel. Indeed, most implementers working with women’s groups assumed that the challenges addressed by their interventions are central to group members’ lives, that women’s groups have ‘natural’ reach and inclusivity by virtue of being community-based, that knowledge imparted will trickle out from facilitator to community members, that people will act upon this knowledge and change long-embedded norms, and that any ‘supply-side’ strengthening carried out is sufficient to address the structural, social and economic barriers to improving health. However, empirical data from our systematic review and a vast social and behaviour change literature suggest that these assumptions are often incorrect. Further, implementers were acutely aware of implementation challenges, especially at scale, and that processes imagined from ‘inputs to outcomes’ were not linear.

We reflected upon the fact that while most women’s groups interventions in India attempted to include and respect most or all the principles in our mid-range theories, they differed greatly in their approaches. This suggests that, while mid-range theories are useful to predict whether a women’s group intervention delivered in one setting or for one issue can be expected to work for others, determining how these principles should be operationalised in each context – called ‘thickening’ the theory with contextual knowledge – is likely to be just as important [34].

## CONCLUSIONS

Women’s groups of different forms and sizes are a central feature of India’s landscape, as well as resources for health in many other countries. We were fortunate to mine a diverse, rich evidence base for insights on how to work with women’s groups to improve health. Evidence overwhelmingly indicates that a commitment to principles of community participation, rather than top-down approaches, is key to identifying feasible, realistic and potentially scalable paths forward. Using our mid-range theory and ‘thickening’ it appropriately for new contexts could help make women’s groups programmes more effective and provide a basis for further implementation research in both India and other settings.

## Additional material


Online Supplementary Document
