# Supplementary material for: Individual interventions, collective lessons: Developing mid-range theory on women’s groups to improve health
Source: J Glob Health. 2024 Aug 16;14:04152. doi: 10.7189/jogh.14.04152 (PMC11327848; doi:10.7189/jogh.14.04152)
Supplement: Online Supplementary Document [file jogh-14-04152-s001.pdf]

Title: Individual interventions, collective lessons: developing mid-range theory on women's groups to improve health

ONLINE SUPPLEMENTARY DOCUMENT

TABLE S1. Mid-range theories by domain

	**Overall**	**Maternal and Newborn Health**	**Nutrition**	**Violence against women**
**Context (In…):**				
**Environmental Context (Context)**	Rural Areas	High child mortality	High levels of maternal and child undernutrition	Domestic violence considered private matter and few women seek help
	Strategic and technical support by an NGO/Govt. (conceptualization, design, staffing, etc.)	Rural areas	Rural areas	Rural and urban areas
		Poor access to health services	Access to nutritious food through markets and public sector	Access to support from the police, health, legal and counselling services are limited or inadequate
		Issue relevant to group participants	Issue relevant to group participants	Strategic and technical support by an NGO/Govt.
		Strategic and technical support by an NGO/Govt.	Strategic and technical support by an NGO/Govt.	
**Intervention Context (Resources):**	Facilitator Characteristics:<br>Trusted by the community<br>Preferably has experiential knowledge<br>Driven and motivated<br>Facilitator and not a teacher<br>Supervision and incentives<br>Training on facilitation and domain	Facilitator characteristics:<br>Trusted and from within the community<br>Experiential knowledge<br>Family support for role<br>Motivated<br>Training in facilitation and domain<br>Facilitates, does not teach	Facilitator characteristics:<br>Trusted and from within the community<br>Experiential knowledge<br>Family support for role<br>Motivated<br>Training in facilitation and domain<br>Facilitates, does not teach	Facilitator characteristics:<br>Trusted and from within the community or group<br>Specifically trained and motivated to address violence<br>Driven and motivated<br>Training in facilitation and domain<br>Facilitates, does not teach

	**Overall**	**Maternal and Newborn Health**	**Nutrition**	**Violence against women**
	Sufficient staff to hold enough groups to cover the population	Supervision & incentives for facilitators	Supervision & incentives for facilitators	Supervision & incentives for facilitators
		Sufficient staff to hold enough group meetings to cover the population	Sufficient staff to hold enough group meetings to cover the population	Intention to address domestic violence but group focus not limited to violence alone
				Groups meet regularly (fortnightly or monthly) for more than 12 months
				Sufficient staff to hold enough group meetings to cover the population
**Mechanism (If…):**				
**Intervention Content & Delivery**	Regular meetings (weekly/fortnightly) with sufficient time devoted to meetings (at least 1-2 hours)	Facilitator actively engages with the most vulnerable population	Interventions to ensure individual-level access (through social norm and behaviour change)	Groups meet regularly and for sufficient time to discuss issue
	Facilitators hold open, non-exclusive meetings with existing or new groups	Facilitator leverages local practices and culture in a positive manner	Facilitators are trained to engage group members in a process of problem identification and solving to address barriers to dietary intake and diversity	Addresses individual attitudes, social norms and behaviour change through discussions on gender, power, and conflict resolution, through non-violent behaviour
	Facilitator goes beyond knowledge transfer and engaged the community in problem-identification and problem-solving	Groups meet regularly and for sufficient time to discuss issue	AND/OR<br>Interventions to ensure household-level direct access to nutritious food (eg: kitchen gardens, agriculture,	Demand-side intervention: Facilitators support group through process of problem identification and developing individual and collective strategies

	Overall	Maternal and Newborn Health	Nutrition	Violence against women
			creches or take home rations)	Facilitators refer survivors to counselling, health, legal and police services and follow them up<br>Facilitator actively engages with the most vulnerable population
	Facilitator leverages local practices and culture in a positive manner		Facilitator leverages local practices and culture in a positive manner	Supply side interventions:<br>Provision of counselling services<br>Capacity building of counselling, health, legal and police service providers<br>Availability of funds to support survivors (from groups, NGO, or govt)
	Facilitator proactively includes vulnerable and marginalized individuals in the intervention		Facilitator actively engages with the most vulnerable population	
			Groups meet regularly and for sufficient time to discuss issue	
**Activities in response to intervention**	Community members for whom the issue is relevant participate in meetings	Community members for whom the issue is relevant participate in meetings	Groups engage in problem-solving and coaching to address individual, household and supply level barriers (including entitlements)	Men willingly involved in the intervention
	Group members engaged in a process of problem identification and solving	Group members engaged in a process of problem identification and solving	Individuals consume additional home grown or externally supplied food	Involvement of mothers in law and/or other family members

	**Overall**	**Maternal and Newborn Health**	**Nutrition**	**Violence against women**
	Groups, communities, and frontline workers take individual or collective action	Groups, communities, and frontline workers take individual or collective action	Community members for whom the issue is relevant participate in meetings	Women participated consistently during the course of the intervention
		Health workers participate in group meeting as members or facilitators	Groups, communities, and frontline workers take individual or collective action	Increase in bystander intervention
				Group members engage in a process of problem identification and solving
				Increase in social support, disclosure and help-seeking from family members, other community members as well as counselling services
				Increased uptake and responsiveness of health, police and legal services
				Community members for whom the issue is relevant participate in meetings
				Groups, communities, and frontline workers take individual and collective action
**Capacities developed in response to the intervention**	Group members provide each other with social support	Group members develop social support	Group members develop social support	Changes in gender roles and attitudes amongst group members
	Group members develop confidence	Group members develop self-confidence	Group members develop self-confidence	Decrease in acceptability of violence in the group and the wider community

	**Overall**	**Maternal and Newborn Health**	**Nutrition**	**Violence against women**
	Increased knowledge about the issue amongst group members	Group members increase their knowledge of perinatal care practices	Group members increase their knowledge of healthy dietary practices	Awareness of services for women
	Members identify problems and solutions,	Greater acceptance among the community and family members for perinatal care practices		Group members develop social support
		Proximal determinants:<br>Better care-seeking for the mother and baby in the perinatal period (if supply is met)<br>Adopting essential newborn care practices		Group members develop self-confidence
**Outcome (Then…):**	Issue needs to be relevant to the group through participatory identification of the problem and/or population-level issue<br>Supply-independent issues/supply-side bottlenecks need to be addressed<br>A priority-setting process should identify 2-3 key issues within a health domain	Reduced neonatal mortality	Improved dietary intake and diversity amongst women and children	Decrease in domestic violence
